# Supplementary material for: Annona crassiflora Mart. Fruit Peel Polyphenols Preserve Cardiac Antioxidant Defense and Reduce Oxidative Damage in Hyperlipidemic Mice
Source: Foods. 2023 May 23;12(11):2097. doi: 10.3390/foods12112097 (PMC10252321; doi:10.3390/foods12112097)
Supplement: Supplementary file 1 [file foods-12-02097-s001.zip › foods-2378596-Supplementary material.pdf]

Table S1. The data presented in Table S1, recently published [1], shows the levels of plasma total cholesterol in different groups of mice, including non-hyperlipidemic mice (naive), tyloxapol-induced hyperlipidemic mice that did not receive any treatment (vehicle), and tyloxapol-induced hyperlipidemic mice treated with different doses (10, 30 or100 mg/kg) of the ethanol extract (CEAc) or polyphenols-rich fraction (PFAc) from *A. crassiflora* fruit peel. The plasma cholesterol levels were measured at several time points: before pre-treatment, after 12 days of pre-treatment, 24 hours after tyloxapol administration, and 48 hours after tyloxapol administration (at euthanasia). The mean value along with the standard error is used to represent the values. The levels of significance are marked with $^{**}p < 0.01$ and $^{***}p < 0.001$, indicating comparison with the vehicle, which was analyzed using two-way ANOVA followed by Dunnett's post hoc test. Adapted from Ramos, et al. [1].

	**Control**		**CEAc**			**PFAc**		
	**Naive**	**Vehicle**	**10 mg/kg**	**30 mg/kg**	**100 mg/kg**	**10 mg/kg**	**30 mg/kg**	**100 mg/kg**
**Before pre-treatment**	32 ± 5	34 ± 3	34 ± 4	33 ± 1	33 ± 2	27 ± 4	27 ± 3	26 ± 3
**After pre-treatment**	36 ± 3	38 ± 4	40 ± 6	40 ± 3	38 ± 2	36 ± 2	30 ± 5	30 ± 3
**After tiloxapol**	36 ± 3***	359 ± 48	242 ± 28	219 ± 60	240 ± 49	382 ± 19	289 ± 62	342 ± 17
**Euthanasia**	60 ± 8**	332 ± 65	391 ± 47	380 ± 61	413 ± 39	391 ± 54	337 ± 89	355 ± 25

Table S2. The data presented in Table S2, recently published [1], shows the levels of plasma triglycerides in different groups of mice, including non-hyperlipidemic mice (naive), tyloxapol-induced hyperlipidemic mice that did not receive any treatment (vehicle), and tyloxapol-induced hyperlipidemic mice treated with different doses (10, 30 or100 mg/kg) of the ethanol extract (CEAc) or polyphenols-rich fraction (PFAc) from *A. crassiflora* fruit peel. The plasma triglyceride levels were measured at several time points: before pre-treatment, after 12 days of pre-treatment, 24 hours after tyloxapol administration, and 48 hours after tyloxapol administration (at euthanasia). The mean value along with the standard error is used to represent the values. The levels of significance are marked with $^{*}p < 0.05$ and $^{**}p < 0.01$, indicating comparison with the vehicle, which was analyzed using two-way ANOVA followed by Dunnett's post hoc test. Adapted from Ramos, et al. [1].

	**Control**		**CEAc**			**PFAc**		
	**Naive**	**Vehicle**	**10 mg/kg**	**30 mg/kg**	**100 mg/kg**	**10 mg/kg**	**30 mg/kg**	**100 mg/kg**
**Before pre-treatment**	56 ± 2	28 ± 2	36 ± 1	37 ± 2	36 ± 2	36 ± 1	37 ± 2	33 ± 2
**After pre-treatment**	65 ± 4	64 ± 4	82 ± 8	86 ± 3	59 ± 2	34 ± 1	30 ± 1	30 ± 5
**After tiloxapol**	65 ± 4**	2990 ± 436	2425 ± 457	2443 ± 621	2095 ± 646	2642 ± 539	2418 ± 250	2794 ± 194
**Euthanasia**	34 ± 5*	1614 ± 112	1520 ± 241	1701 ± 299	1306 ± 696	917 ± 287	1344 ± 409	1079 ± 196

Table S3. The levels of fecal total lipids, triglycerides, and cholesterol (measured in mg/g) were examined in various groups of mice (adapted from Ramos, et al. [1]). These included non-hyperlipidemic mice, non-treated hyperlipidemic mice induced by tyloxapol (referred to as "vehicle"), and hyperlipidemic mice induced by tyloxapol that were treated with either 10, 30, or 100 mg/kg of an ethanol extract (CEAc) or polyphenols-rich fraction (PFAc) from the fruit peel of *A. crassiflora*. The measurements were taken both before and after the administration of tyloxapol, with a 12-day pre-treatment period before the administration of tyloxapol. The levels of significance are marked with $*p < 0.05$ and $**p < 0.01$, indicating comparison with the vehicle, and $\#p < 0.05$, $\#\#p < 0.01$, and $\#\#\#p < 0.001$, indicating comparison between before and after tyloxapol administration (two-way ANOVA followed by Dunnett's post hoc test).

		**Total lipids (mg/g)**		**Triglycerides (mg/g)**		**Total cholesterol (mg/g)**	
		**Before**	**After**	**Before**	**After**	**Before**	**After**
**Control**	**Naive**	13 ± 2	14 ± 3	5 ± 0	$5 \pm 0^{***}$	4 ± 0	$4 \pm 0^{***\#\#}$
	**Vehicle**	13 ± 1	$23 \pm 2^{\#\#}$	5 ± 0	$7 \pm 0^{\#\#}$	4 ± 0	$5 \pm 0^{\#\#\#}$
**CEAc**	**10 mg/kg**	10 ± 1	14 ± 3	4 ± 0	$6 \pm 0^{**\#\#\#\#}$	$4 \pm 0^{**}$	$5 \pm 0^{\#\#\#}$
	**30 mg/kg**	13 ± 3	17 ± 3	5 ± 0	$7 \pm 1^{\#\#}$	4 ± 0	$6 \pm 0^{\#\#\#}$
	**100 mg/kg**	12 ± 2	16 ± 4	5 ± 0	$6 \pm 0^{*\#\#\#\#}$	$4 \pm 0^{**}$	$5 \pm 0^{\#\#\#}$
**PFAc**	**10 mg/kg**	10 ± 1	13 ± 4	5 ± 0	$7 \pm 0^{\#\#}$	5 ± 0	$6 \pm 0^{\#}$
	**30 mg/kg**	11 ± 1	15 ± 3	5 ± 0	$7 \pm 0^{\#\#\#}$	5 ± 0	$5 \pm 0^{**}$
	**100 mg/kg**	$9 \pm 1^{*}$	14 ± 4	5 ± 0	$7 \pm 0^{\#\#\#}$	4 ± 0	$5 \pm 0^{\#}$

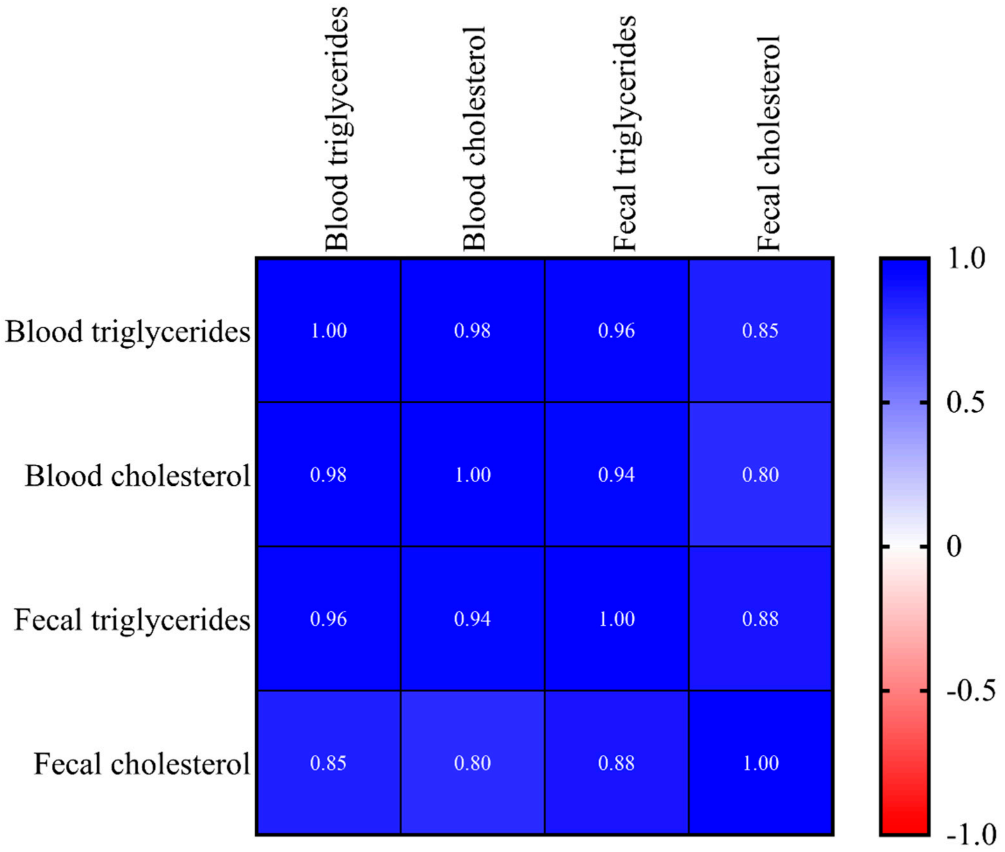

Figure S1. Pearson's correlation analysis between blood triglycerides, blood total cholesterol, fecal triglycerides and fecal total cholesterol.

Table S4. Pearson's correlation analysis between blood triglycerides (TG), blood total cholesterol (TC), fecal triglycerides (TG) and fecal total cholesterol (TC).

**Blood and fecal lipid parameters – Pearson's correlation**					
Blood TG x Blood TC	Blood TG x Fecal TG	Blood TG x Fecal TC	Blood TC x Fecal TG	Blood TC x Fecal TC	Fecal TG x Fecal TC
$r = 0.978$	$r = 0.956$	$r = 0.849$	$r = 0.938$	$r = 0.805$	$r = 0.883$
$p < 0.001$	$p < 0.001$	$p < 0.001$	$p < 0.001$	$p < 0.001$	$p < 0.001$

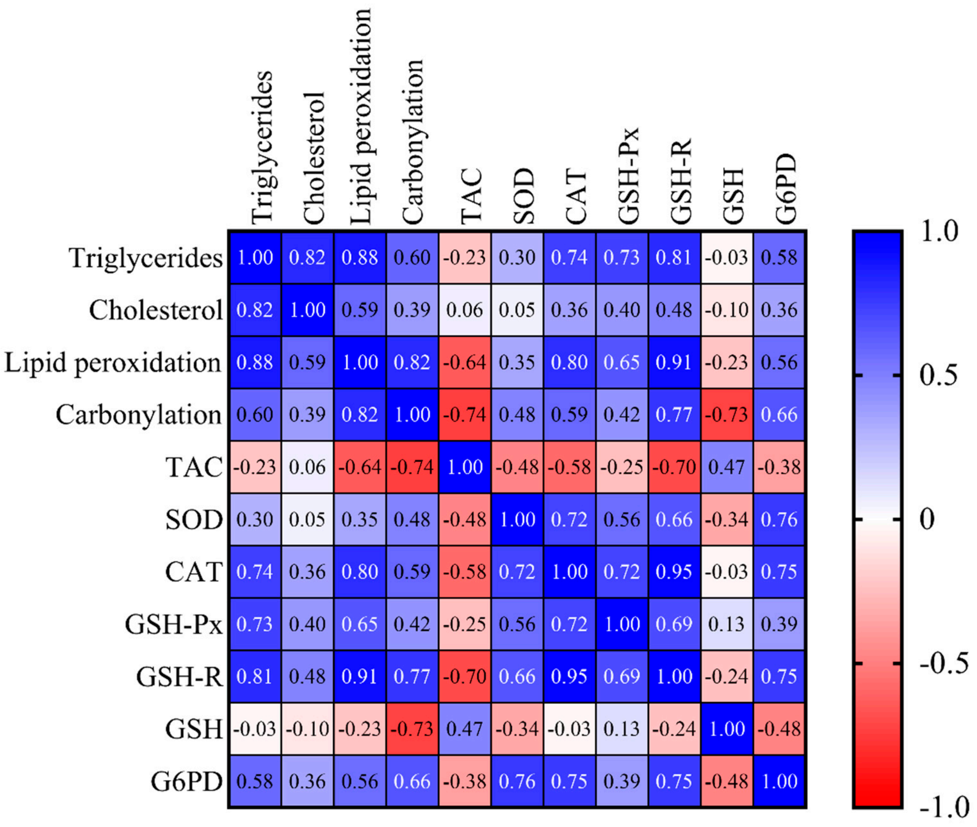

Figure S2. Pearson's correlation analysis between blood triglycerides, blood cholesterol and oxidative stress parameters in heart tissue (lipid peroxidation, protein carbonylation, total antioxidant capacity (TAC), activities of superoxide dismutase (SOD), catalase (CAT), glutathione peroxidase (GSH-Px), glutathione reductase (GSH-R), glucose 6-phosphate dehydrogenase (G6PD), and reduced glutathione content (GSH)).

Table S5. Pearson's correlation analysis between blood triglycerides (TG), blood total cholesterol (TC) and oxidative stress parameters in heart tissue (lipid peroxidation (LP), protein carbonylation (PC), total antioxidant capacity (TAC), activities of superoxide dismutase (SOD), catalase (CAT), glutathione peroxidase (GPx), glutathione reductase (GR), glucose 6-phosphate dehydrogenase (G6PD), and reduced glutathione content (GSH). Only correlations with *p*-values less than 0.05 are shown.

**Blood lipid parameters and cardiac oxidative stress parameters – Pearson's correlation**		
Blood TG x Blood TC	$r = 0.822$	$p = 0.012$
Blood TG x LP	$r = 0.880$	$p = 0.004$
Blood TG x CAT	$r = 0.741$	$p = 0.035$
Blood TG x GPx	$r = 0.727$	$p = 0.041$
Blood TG x GR	$r = 0.806$	$p = 0.016$
LP x PC	$r = 0.819$	$p < 0.001$
LP x CAT	$r = 0.796$	$p = 0.018$
LP x GR	$r = 0.914$	$p = 0.001$
PC x TAC	$r = -0.738$	$p = 0.037$
PC x GR	$r = 0.770$	$p = 0.027$
PC x GSH	$r = -0.728$	$p = 0.04$
TAC x GR	$r = -0.696$	$p = 0.05$
SOD x CAT	$r = 0.725$	$p = 0.042$
SOD x G6PD	$r = 0.764$	$p = 0.027$
CAT x GPx	$r = 0.720$	$p = 0.044$
CAT x GR	$r = 0.954$	$p < 0.001$

CAT x G6PD	$r = 0.752$	$p = 0.031$
GPx x G6PD	$r = 0.390$	$p < 0.001$
GR x G6PD	$r = 0.755$	$p = 0.030$

Reference

[1] L.P.A. Ramos, A.B. Justino, N. Tavernelli, A.L. Saraiva, R.R. Franco, A.V. de Souza, H.C.G. Silva, F.B.R. de Moura, F.V. Botelho, F.S. Espindola, Antioxidant compounds from Annona crassiflora fruit peel reduce lipid levels and oxidative damage and maintain the glutathione defense in hepatic tissue of Triton WR-1339-induced hyperlipidemic mice, Biomedicine & pharmacotherapy = Biomedecine & pharmacotherapie 142 (2021) 112049.
